# Multilevel predictors of adolescent physical activity: a longitudinal analysis

**DOI:** 10.1186/1479-5868-9-8

**Published:** 2012-02-06

**Authors:** Mary O Hearst, Carrie D Patnode, John R Sirard, Kian Farbakhsh, Leslie A Lytle

**Affiliations:** 1 Division of Epidemiology and Community Health, University of Minnesota, 1300 South Second Street, Suite 300, Minneapolis, MN 55454, USA; 2 Kaiser Permanente Center for Health Research, Portland, OR, USA; 3 Curry School of Education & Kinesiology, University of Virginia, Charlottesville, VA, USA

**Keywords:** Adolescent, Multilevel, Predictors of physical activity, Longitudinal

## Abstract

**Background:**

To examine how factors from a social ecologic model predict physical activity (PA) among adolescents using a longitudinal analysis.

**Methods:**

Participants in this longitudinal study were adolescents (ages 10-16 at baseline) and one parent enrolled in the Transdisciplinary Research on Energetics and Cancer-Identifying Determinants of Eating and Activity (TREC-IDEA) and the Etiology of Childhood Obesity (ECHO). Both studies were designed to assess a socio-ecologic model of adolescent obesity risk. PA was collected using ActiGraph activity monitors at two time points 24 months apart. Other measures included objective height and weight, adolescent and parent questionnaires on multilevel psychological, behavioral and social determinants of PA, and a home PA equipment inventory. Analysis was conducted using SAS, including descriptive characteristics, bivariate and stepped multivariate mixed models, using baseline adjustment. Models were stratified by gender.

**Results:**

There were 578 adolescents with complete data. Results suggest few statistically significant longitudinal associations with physical activity measured as minutes of MVPA or total counts from accelerometers. For boys, greater self-efficacy (B = 0.75, *p *= 0.01) and baseline MVPA (B = 0.55, *p *< 0.01) remained significantly associated with MVPA at follow-up. A similar pattern was observed for total counts. For girls, baseline MVPA (B = 0.58, *p *= 0.01) and barriers (B = -0.32, *p *= 0.05) significantly predicted MVPA at follow-up in the full model. The full multilevel model explained 30% of the variance in PA among boys and 24% among girls.

**Conclusions:**

PA change in adolescents is a complex issue that is not easily understood. Our findings suggest early PA habits are the most important predictor of PA levels in adolescence. Intervention may be necessary prior to middle school to maintain PA through adolescence.

## Background

The U.S. Department and of Health and Human Services recommends that children and adolescents engage in 60 min or more of physical activity (PA) every day, with most of that time in moderate- to vigorous-intensity [1]. Engagement in moderate-to-vigorous physical activity (MVPA) typically decreases as adolescents move through their teen years. Examining data from a large adolescent cohort, Laska et al. showed that MVPA among girls declined from 5.9 h/week during the transition from early adolescence to mid-adolescence with a further decline to 3.5 h/week by late adolescence [2]. A similar, but less pronounced decline was also seen in boys. It is known that PA is inversely associated with cardiovascular disease risk factors during adolescence and into adulthood; [3-5] therefore, identifying and subsequently intervening upon factors that help predict and promote adolescent PA over time is crucial for long-term health outcomes.

There have been several review articles in the past decade describing the correlates of PA among adolescents [3,6-8]. The reviewers generally conclude that the state of the science is limited due to the complexity of the issue, limited external validity, and the lack of measurement precision. Even studies using the most complex multilevel models only explain a low percentage (5.3-5.7%) of the variance in PA levels among children and adolescents [3]. There are relatively few longitudinal and intervention studies that help to describe potential causal factors or determinants of change in PA [9,10].

What is generally agreed upon is that no one factor explains PA levels among adolescents or predicts the decline in PA as children age, but that a reasonable approach is to examine multilevel influences PA. Social-ecological models [11] attempt to define the complexity of behavioral choices and typically include psychological, behavioral, social, home, school, and neighborhood environmental factors. Non-modifiable influences, such as demographic characteristics, are informative as the findings may highlight the need to target intervention strategies or develop differential intervention strategies based on the composition of the target group. An ecological model used to examine levels of PA in youth might reasonably examine intrapersonal factors (for example, attitudes and opinions youth hold about being active), their own behavioral experiences (for example, the extent to which being physically active is part of their behavioral routine), their social environment (for example the types of role models and encouragement that they receive from others to be active) and factors in the physical environments of their neighborhoods (such as how safe it is to play outside or the availability of parks and recreational areas) A social ecological model would suggest that all of these factors hold some importance in understanding PA behavior and that the factors are inter-related.

Previously, we published the influences of individual, social and environmental correlates of PA using cross-sectional data [12]. Given the paucity of high-quality longitudinal research, it was important for us to examine these relationships longitudinally. Therefore, the purpose of this manuscript was to assess change in PA over time in a large sample of adolescents. This adds to the limited literature in two ways. First, the longitudinal nature of the data in the adolescent cohort allowed us to examine how early exposures predict later levels of PA and second, the use of multilevel and multi-factorial models allowed us to simultaneously identify individual, social, and environmental (i.e., home, school, and neighborhood) predictors of change in adolescent PA. The hypothesis was that factors at each level would be predictive of MVPA and that the predictors would differ by gender.

## Methods

### Study design

The data from this study came from two longitudinal studies comprised of adolescents (ages 10-16 at baseline) and one of their parents: the Transdisciplinary Research on Energetics and Cancer - Identifying Determinants of Eating and Activity (TREC-IDEA) and Etiology of Childhood Obesity (ECHO) study. Both studies were designed to examine the social and environmental influences on unhealthy weight gain in adolescents [13]. A social ecological model was developed to guide the research and included contextual factors from the intrapersonal and social and physical environments, their impact on weight related behaviors including levels of PA, and the impact of the contextual factors and behaviors on body composition [13]. The TREC-IDEA and ECHO studies used identical data collection instruments and measurement protocol and recruited from the same target population. Both studies were approved by the University of Minnesota Institutional Review Board.

For the TREC-IDEA study, youth were recruited from a preexisting cohort, [14] a permit application listing from the Minnesota Department of Motor Vehicles, and a convenience sample from the St. Paul-Minneapolis metropolitan area. Baseline data collection (n = 349 adolescent/parent dyads) began in October, 2006, and concluded in May, 2007. Annual data collection was timed for each participant such that the measurements were taken at the same time of year for 24 month follow-up. Ninety-four percent of the baseline sample (n = 328) was re-measured 24-months post baseline.

For the ECHO study, 374 youth and a parent were recruited from the membership of Health Partners (HP) health plan within the seven-county metropolitan area of Minneapolis, St. Paul, Minnesota between June 2007 and March 2008. We used a recruitment procedure that targeted a range of overweight and healthy weight youth and parents and that oversampled minorities. To be eligible for enrollment, adolescents were required to be current HP members, in grades 6th to 11th in the fall of 2007, residing in one of the randomly selected middle or high-school districts included in the sample, have a parent willing to participate and be willing to allow their names and contact information to be sent from HP to the study team at the University of Minnesota for further eligibility screening, consent and measurement. Baseline data collection (n = 374 adolescent/parent dyads) began in 2007; there was one additional data collection period in 2009. Eighty-six percent of the sample was assessed in both time periods.

In both TREC/IDEA and ECHO, parent/adolescent dyads were excluded from eligibility if they planned to move from the area in the next 3 years, had a medical condition that affected their growth, were non-English speaking or otherwise had difficulty comprehending English, or had any other physical or emotional condition that would affect their diet/activity levels or make it difficult to complete measurements. Loss to follow-up for participants in both studies was due to participants not having time to participate (IDEA = 19; ECHO = approximately 45); moved (IDEA = 2; ECHO = approximately 3); and unable to connect (ECHO = approximately 4). The Institutional Review Board at the University of Minnesota approved both studies.

### Measures

Individual-level data collection occurred at the Epidemiology Clinical Research Center (ECRC) of the University of Minnesota by trained staff. Data collection included measured height, weight and body composition and self-report survey data by adolescents and parents. Parents completed a home-based physical activity equipment survey and we used Geographical Information System (GIS) software to assess elements of the neighborhood physical environment. Details of all measures are provided below.

#### Dependent variable: physical activity

The ActiGraph activity monitor, model 7164 (ActiGraph, LLC, Pensacola, FL) was used to collect 7 days of PA data using 30-s epochs (data collection intervals). The monitor is an objective measure of PA and has been previously validated for use with children in laboratory and field settings [15-17]. At monitor distribution, trained research staff fit the monitor to each student and provided the students with written and verbal instructions for wearing the monitor and for sending it in to study staff upon completion of the 7 days of wearing.

A custom developed software program was created by one of the authors (JRS) using Visual Basic (version 6.0, Microsoft, Corp) and modified for the current study design [18,19]. Daily inclusion criteria were established to determine days and times with acceptable accelerometer data. Blocks of time incorporating at least 30 continuous minutes of "0" output were considered to be times when the subject was not wearing the monitor and were eliminated. Missing data within an adolescent's 7-day record were replaced via imputation based on the Expectation Maximization (EM) algorithm [20]. On average, approximately 22 h of data (about 13%) were imputed over the 7 days of data collection. Summary PA variables were calculated using the Freedson age-specific count cutoffs [21] distinguishing moderate and vigorous intensity based on age-adjusted MET values [22,23].

#### Individual level measures: covariates

Pubertal status was assessed by the self-report Pubertal Development Scale (PDS) [24]. The PDS is a five question summed score with good internal consistency (alpha = 0.77) and good correlation with physician rating (0.61-0.67) [24]. Puberty was included as a covariate because different stages of pubertal development, particularly among girls, have been shown to be related to participation in PA and PA decline [25]. Demographic and socioeconomic status collected from the parents included whether the adolescent receives free or reduced cost lunch (1 = yes, 0 = no) and highest household level of parent education (1 = college or higher, 0 = less than college) and from the adolescent included age, gender (1 = female, 0 = male), and race (1 = white, 0 = other).

Adolescent height, weight and percent body fat (PBF) were measured by trained staff with the adolescent wearing a T-shirt and a pair of shorts. Height was measured using a Shorr Height Board to the nearest 0.1 cm, while the participants stood in their bare feet. Body mass and total body fat was determined using a digital bioelectrical impedance scale (Tanita TBF-300A Body Composition Analyzer/Scale, Tanita Corporation, Tokyo, Japan). Because previous findings with these data showed that percent body fat was more highly correlated with PA than was BMI, [26] we chose to include only percent body fat in the models. Weight status has been shown to be associated with lower levels of motivation to engage in PA [27], thus weight status could confound the relationship of PA change over time.

#### Potential predictors: intrapersonal factors

*Self-efficacy *was self-reported by adolescents using a previously tested scale [28]. The scale consisted of eight questions that gauged children's confidence in their ability to overcome barriers and seek support in order to be active. Responses were on a 5-point scale from 1 *(strongly disagree) *to 5 *(strongly agree)*. Internal consistency of this scale was α = 0.82. Self-efficacy has been shown to correlate with PA in children and also mediate the relationship between social support by parents and peers [29-31].

*PA enjoyment *was measured using seven questions with the stem of "When I am active..." followed by items such as "I feel bored," and "I dislike it" [28]. Responses were measured on a 5-point scale from 1 (*strongly disagree) *to 5 (*strongly agree) *with a higher score indicative of more enjoyment related to PA. Internal consistency of this scale was α = 0.94. Models of behavior change suggest that behavior is more likely to occur if positive outcomes are expected, such as enjoyment of PA [32], and is supported in the literature [33].

*Perceived barriers *to PA was assessed with 12 items adapted from Dishman et al. [34] Items such as "I don't like to sweat" and "It would make me embarrassed" were used to identify potential obstacles that kept the adolescent from being physically active. Items were rated on a 5-point scale, from 1 (*never) *to 5 (*very often)*. A higher score reflected perceiving more barriers to PA, with the internal consistency α = 0.83. Contrary to PA enjoyment, perceived barriers related to PA are associated with less PA among adolescents [35].

#### Potential predictors: behavioral repertoire

Daily minutes of screen time was assessed as part of the self-report survey. Weekday screen time behavior was assessed asking: "On a typical weekday (Monday-Friday), how many hours do you spend watching TV?" The same question was asked for watching DVDs or videos, Nintendo/Play Station/computer games and internet/computers. A similar question was used to assess weekend (Saturday-Sunday) screen time behavior [2,36]. Six response options ranged from "none" to "6+ hours" per day. Responses categories were set at the mid-range, weighted for weekday versus weekend, summed and divided by seven resulting in the number of daily minutes of screen time behavior. Sedentary behavior has a mixed relationship with PA as seen in a latent class analysis of PA and sedentary behavior [37]. Therefore, we included sedentary behavior as a potential confounder.

Sports team participation was measured by a self-report survey question asking, "How many team or individual sports or activities such as varsity or junior varsity sports, intramurals, or out-of-school programs/activities do you currently participate in" [38,39]? Response categories were recorded to reflect yes or no to team sport participation. Differences in activity levels may be due to sports participation [40], particularly when considering healthy and overweight adolescents.

#### Potential predictors: social environment

*Perceived parent support *and *perceived peer support *scales were self-reported on the survey by the adolescents. Adolescents indicated how often during a typical week their mother or father or one of their friends provided support related to PA. Items were scored on a scale from 1 (*never*) to 5 (*every day*) and included statements such as "encouraged you to do physical activities," and "watched you participate." Internal consistency of these scales were also good at α = 0.76 and 0.86 for parent and peer social support, respectively [41]. Social support plays an important role in adolescent PA [12].

#### Potential predictors: the physical environment. Home and neighborhood

The *home PA environment *was evaluated using a validated self-reported instrument, the Physical Activity and Media Inventory (PAMI), completed by the parents at home [42]. The intent of the PAMI was to capture both availability and accessibility of home based equipment that may support PA or sedentary behavior. The inventory included a list of 42 PA equipment items and 5 media equipment items. For each room within the home, parents were asked to indicate specific quantities and accessibility of each particular piece of equipment. A PA availability and accessibility score was created which reflects the product of each item quantity and accessibility. A higher score reflected greater presence and access of PA equipment at home. Parents received the PAMI during the clinic visit with instructions to return the form after completion. The home environment is associated physical activities among adolescents, with differences noted between boys and girls [43].

Two survey scales were used to characterize adolescent perceptions of the neighborhood environment. *Perceived neighborhood safety *and *ease of mobility *were based on items included in the Neighborhood Environment Walkability Scales (NEWS) which indicate high test-retest reliabilities among adults from neighborhoods with differing levels of "walkability" [44]. Perceived neighborhood safety was measured with five items on the adolescent survey on a 4-point scale ranging from 1 (*strongly disagree*) to 4 (*strongly agree*). Examples of items included, "It is safe to walk or play in my neighborhood during the day." Responses were recorded for consistency in direction such that higher scores reflected less perceived safety, or more safety concerns. The internal consistency was α = 0.75. The perceived ease of mobility scale measured adolescent's perceptions of how easy or difficult it was to navigate their neighborhood by walking and/or biking. A sample question from the five item scale was "There are sidewalks on most of the streets in my neighborhood". Response categories ranged from 1 (*strongly disagree*) to 4 (*strongly agree*, with a high value reflected better ease of access on foot or bicycle. Reliability for the ease of mobility scale was α = 0.78.

Finally, we calculated a 'walkability index' from three variables available from the Geographic Information Systems (GIS) software package, ArcGIS, version 9.2 (Environmental Systems Research Institute, Redlands, CA) including residential, intersection and employment density. Using the participant home address, residential density was calculated as the number of persons in housing units per unit of land area excluding water. Intersection density provided a measure of street connectivity with higher connectivity providing more direct routes for pedestrians. It was calculated as the number of street intersections per unit of land area with interstate highways removed. Employment density was calculated as the total employees per of land area excluding water. A three-component *walkability index *was created to characterize the built environment patterns conducive to active transit around each home by calculating the normalized distribution (z-score) of the three measures and summing the three variables [45]. A higher score of the walkability index reflect greater ease of active transit to locations in the neighborhood, which have been shown to be associated with PA among adolescents [12,46]. A recent review of the neighborhood environment and physical activity among youth showed the most supported correlates for adolescents were walkability, traffic speed/volume, access to recreation facilities and other features of the build environment such as residential density [47].

### Statistical methods

As the nature of PA differs between boys and girls, [12] we stratified all analyses a priori and present gender specific estimates for all analyses, using SAS v. 9.1 for Windows (Cary, NC: SAS Institute Inc). Given the identical protocols and measurements, data for the IDEA and ECHO cohort studies were combined for analyses. Descriptive statistics included calculating proportions and variable distributions for the entire sample, gender stratified and using chi-square and *t*-test statistics to determine statistical differences by gender. We used baseline variables to predict PA at 24-month follow-up, adjusting for baseline PA. We used a stepped approach to statistical modeling, generalized estimating equations, using the procedure PROC GENMOD, with school indicated as the random effect given that some youth were nested in school (average = 2.5 students per school). We tested for differences between IDEA and ECHO participants on demographic factors (*t*-test and chi-square) and conducted post-hoc regression modeling with each study sample as described above.

## Results

Table 1 presents the sample characteristics for the analytic sample. The ECHO sample was younger, lower income, more racial/ethnic diversity and had a higher proportion overweight compared to the IDEA sample. Given the samples were drawn from the same target population and completed the same measurement protocols; this difference adequately enhanced the generalizability of the study. There were 578 adolescents included in the analysis from baseline, with an even gender split. The sample was predominantly white, college-educated and 11% received free or reduced cost lunch. Adolescents spent over 5 h per day in front of a screen, and 31 min per day engaged in MVPA. Psychosocial factors and social and physical environment scales and variables had central tendency with good distribution. See Table 1.

**Table 1:** Sample Characteristics, TREC IDEA and ECHO, 2006-2010

	All (n = 578)			Boys (n = 287)		Girls (n = 291)		*P*-value
	**Mean**	**SD**	**Range**	**Mean**	**SD**	**Mean**	**SD**	

Percent White	86.9	33.8		88.5	32.0	85.2	35.5	0.20

Household education, (col grad/prof training)	78.0	41.4		80.5	39.7	75.6	43.0	0.18

Percent free/reduced lunch	10.6	30.8		9.8	29.7	11.3	31.8	0.61

Age	14.6	1.8		14.5	1.8	14.6	1.8	0.91

Pubertal Status	2.9	0.8		2.5	0.7	3.2	0.7	< 0.01

%Body fat	20.9	9.9	3.6-60.1	16.0	8.9	25.9	8.3	< 0.01

% Participate in one or more team now and last year	64.9	47.8	0.0-100.0	65.2	47.7	64.6	47.9	0.89

Daily minutes of screen time	312.4	217.5	8.6-1311.4	345.5	231.9	279.8	197.4	< 0.01

Daily minutes of moderate to vigorous physical activity	30.9	17.1	2.2-145.4	35.1	18.9	26.7	14.1	< 0.01

PA Self Efficacy	30.8	4.8	11.0-40.0	31.3	4.4	30.2	5.1	0.01

PA Enjoyment	29.8	5.3	7.0-35.0	30.6	4.9	29.0	5.5	< 0.01

PA Barriers	22.6	6.7	12.0-49.0	21.1	5.9	24.1	7.1	< 0.01

PA Parental Support	11.4	3.7	4.0-20.0	11.5	3.5	11.3	3.8	0.43

PA Peer Support	11.5	4.0	4.0-20.0	11.7	3.9	11.3	4.1	0.18

PA equipment Availability & Access Summary Score	247.4	141.2	0.0-1012.0	272.0	151.6	223.0	125.8	< 0.01

Perceived neighborhood safety	8.6	2.4	5.0-17.0	8.4	2.4	8.8	2.3	0.07

Perceived walking infrastructure quality	13.7	2.9	5.0-20.0	13.7	2.9	13.7	3.0	0.91

GIS Walkability Index	0.1	2.4	-4.1-11.4	0.1	2.3	0.1	2.5	0.85

Gender differences in baseline values included girls being more advanced on the pubertal scale, having more body fat, engaging in less screen time and less MVPA, enjoying PA less and reporting more PA barriers and less PA equipment available in the homes compared to boys.

Daily minutes of MVPA at baseline was 31 min and significantly increased to 37 min at follow-up. This increase was seen for boys (baseline = 35 min; follow-up = 41 min) and girls (baseline = 27 min; follow-up = 32 min). The average minutes of total activity per day (light, moderate and vigorous) decreased significantly for the whole sample (16.3 min) and decreased by 16 min for both boys and girls, driven by a decrease in light activity levels. Pearson correlation coefficients between baseline and follow-up were statistically significant (*p *≤ 0.001) for the total sample (r = 0.46), boys (r = 0.47) and girls (r = 0.38).

Table 2 presents the regression results for predictors of change in daily minutes of MVPA among boys. Model 1 explores the role of baseline sociodemographic, body composition and levels of MVPA at baseline as predictors of PA 24 months later and finds that MVPA at baseline and age are significantly related to MVPA at 24 months. Model 2 adds the intrapersonal and behavioral factors showing an additional significant relationship between self-efficacy and PA at 24-months follow-up. Model 3 adds elements of the social environment with no substantial change noted. Finally, model 4 adds the features of the physical environment again resulting in no additional changes in significant predictors.

**Table 2:** Predictors of physical activity measured as average mean minutes of daily moderate to vigorous physical activity among adolescent boys, TREC IDEA and ECHO, 2006-2010

N = 287												
**Variable**	**Model 1**			**Model 2**			**Model 3**			**Model 4**		

**Parameter**	**Coeff**	**SE**	***P*-value**	**Coeff**	**SE**	***P*-value**	**Coeff**	**SE**	***P*-value**	**Coeff**	**SE**	***P*-value**

MVPA baseline	0.579	0.086	< .0001	0.564	0.095	< .0001	0.554	0.095	< .0001	0.547	0.099	< .0001

Study	-0.552	2.500	0.825	-0.520	2.614	0.842	-0.695	2.580	0.788	-1.074	2.702	0.691

White	3.785	4.623	0.413	3.072	4.660	0.510	3.211	4.606	0.486	3.866	4.557	0.396

College education	-1.234	2.721	0.650	-0.402	2.843	0.888	-0.337	2.792	0.904	-0.568	3.030	0.852

Free/reduced lunch	1.976	3.725	0.596	1.498	3.290	0.649	1.174	3.443	0.733	0.285	3.694	0.938

Age of student	3.150	1.208	0.009	3.296	1.175	0.005	3.132	1.184	0.008	3.268	1.181	0.006

Puberty	-3.265	2.847	0.251	-3.638	2.825	0.198	-3.752	2.814	0.182	-4.013	2.851	0.159

% Body Fat	-0.019	0.102	0.855	-0.013	0.109	0.907	-0.008	0.109	0.943	-0.063	0.126	0.616

PA Self-efficacy				0.719	0.272	0.008	0.680	0.276	0.014	0.752	0.279	0.007

PA enjoyment				0.070	0.246	0.776	0.069	0.244	0.778	0.013	0.247	0.957

PA barriers				0.047	0.231	0.839	0.037	0.227	0.870	-0.004	0.231	0.986

Daily min screen time				-0.001	0.004	0.801	-0.002	0.004	0.714	-0.002	0.004	0.692

Sport team participation				-3.105	2.464	0.208	-3.383	2.749	0.218	-3.506	2.813	0.213

PA: Parent support							-0.224	0.431	0.602	-0.246	0.422	0.561

PA Peer support							0.314	0.310	0.311	0.299	0.309	0.333

PA equipment Availability & Access Summary Score										0.004	0.011	0.718

Safety concerns										0.766	0.568	0.178

Ease of mobility										-0.063	0.475	0.895

Walkability Index										0.361	0.575	0.530

For boys, three variables consistently emerge as predictors of MVPA. The most consistent, positive and significant predictor of MVPA at the follow-up measurement time across the four models was MVPA at baseline. The age at baseline measurement was also a consistent, positive and significant predictor of MVPA across the four models. Finally, PA self-efficacy remains a statistically significant predictor of PA at 24 months in the full model with baseline levels of self-efficacy in boys predicting subsequent levels of MVPA. The amount of variance explained in the full model for PA for boys was r^2 ^= 0.30.

Data for girls are presented in Table 3. The full model shows that baseline MVPA and the age of the girls at baseline were positively and significantly associated with higher levels of MVPA at 24 months. In addition, earlier pubertal development was associated with lower levels of MVPA in the final model at the follow-up period. Of the intrapersonal, social and physical environmental factors examined, only baseline perceptions of barriers related to being physically active were significantly and inversely related to PA at the follow-up period. The amount of variance explained in the full model for girls was r^2 ^= 0.24.

**Table 3:** Predictors of physical activity measured as average mean minutes of daily moderate to vigorous physical activity among adolescent girls, TREC IDEA and ECHO, 2006-2010

N = 291												
**Variable**	**Model 1**			**Model 2**			**Model 3**			**Model 4**		

**Parameter**	**Coeff**	**SE**	***P*-value**	**Coeff**	**SE**	***P*-value**	**Coeff**	**SE**	***P*-value**	**Coeff**	**SE**	***P*-value**

MVPA baseline	0.527	0.199	0.008	0.536	0.212	0.011	0.536	0.211	0.011	0.572	0.214	0.007

Study	4.263	2.898	0.141	3.610	2.683	0.179	3.751	2.750	0.173	4.550	2.843	0.110

White	0.719	2.750	0.794	1.282	3.114	0.681	1.032	3.056	0.736	0.524	2.739	0.848

College education	1.208	2.134	0.572	0.320	2.246	0.887	0.356	2.278	0.876	-0.485	2.122	0.819

Free/reduced lunch	1.877	3.383	0.579	2.361	3.607	0.513	2.577	3.563	0.469	2.380	3.911	0.543

Age of student	3.377	0.773	< .0001	3.232	0.745	< .0001	3.369	0.798	< .0001	3.751	0.808	< .0001

Puberty	-5.007	2.734	0.067	-4.967	2.623	0.058	-4.854	2.596	0.062	-5.709	2.596	0.028

% Body Fat	-0.050	0.129	0.697	-0.065	0.133	0.626	-0.071	0.134	0.598	-0.007	0.133	0.958

PA Self-efficacy				-0.155	0.208	0.456	-0.183	0.218	0.401	-0.293	0.213	0.169

PA enjoyment				-0.103	0.247	0.676	-0.094	0.246	0.702	-0.060	0.262	0.820

PA barriers				-0.332	0.168	0.048	-0.319	0.164	0.052	-0.315	0.160	0.049

Daily min screen time				-0.007	0.005	0.162	-0.006	0.005	0.192	-0.006	0.005	0.246

Sport team participation				-6.105	4.300	0.156	-6.222	4.560	0.172	-6.263	4.964	0.207

PA: Parent support							0.297	0.402	0.459	0.167	0.410	0.683

PA Peer support							-0.155	0.320	0.629	-0.145	0.319	0.650

PA equipment Availability & Access Summary Score										0.015	0.013	0.231

Safety concerns										0.247	0.513	0.630

Ease of mobility										0.021	0.416	0.959

Walkability Index										0.188	0.551	0.733

Post-hoc analysis was conducted modeling the IDEA and ECHO samples independently and stratified by gender. The findings were comparable with few exceptions. Among boys, the combined sample showed a significant relationship with age, but the study specific analysis showed an age effect only among IDEA participants and a relationship between change in PA and more safety concerns. This is consistent with the observed decrease in light physical activity over time. Self-efficacy and baseline PA remained significant across both samples of boys. Among girls, age, baseline PA and puberty remained significantly associated with PA at follow-up. Barriers and self-efficacy were associated with PA at follow-up among girls in the ECHO sample, but not the IDEA sample, which is also consistent with the older mean age of the IDEA sample.

## Discussion

Our longitudinal analysis, which included the IDEA sample and adds the ECHO sample, provided another perspective examining the longitudinal relationship between early exposures and subsequent PA levels. In our longitudinal analysis, for both boys and girls, the most powerful predictor of PA at 24 months was baseline levels of PA. This is important as it re-emphasizes that many health behaviors are established early in life and become habituated. Interventions should focus on establishing strong lifestyle habits of PA prior to or in early adolescence to ameliorate the typical decline during adolescence into young adulthood. Gaining a better understanding of the forces at play that cause some children to increase activity while others to decline is key in intervening at the most appropriate age. For example, a recent longitudinal analysis showed that MVPA decreased significantly from age 9 to 15, but the linear rate of decline leveled off around age 15 [48]. In addition, developing effective interventions at the family and community level that help foster early, enjoyable experiences with PA is an important public health goal.

Early experience with physical activity opportunities may influence how an individual feels about being active and if they self-define as an active person. Those perceptions may impact activity long term. For example, if an adolescent experiences low levels of self-efficacy, enjoyment and high perceptions of barriers to being active when they are younger, they may decide that being physically active is not a good option for them and that perception may persist. However, it has been found that such perceptions can be altered via targeted intervention [49]. In addition, physical activity requires some skills. If skills are not learned and practiced during early adolescent (behavioral repertoire), becoming active later in adolescence without an external intervention may be more difficult or frustrating and be an additional barrier to being active.

Among boys, the final models suggested that baseline self-efficacy or confidence to be physically active was an important predictor of positive change in PA over time; self-efficacy was a statistically significant correlate of PA in our cross-sectional analysis as well [13]. Self-efficacy is among the only consistently positively associated variables with MVPA among children and adolescents [9] and has been found to mediate changes in PA in several intervention studies among youth [10]. It is plausible that the younger a child is when they become involved in activity and experience success (as measured through peer approval, tangible rewards, or parental recognition) the more confident they become - leading to maintenance of activity over time. The nature and types of physical activities that preadolescent and adolescent boys often participate in (e.g., both free time "play" and organized sports) require a certain level of motivation and persistence, of which confidence is a key driver.

For girls, the construct of perceived barriers was the strongest predictor of change in PA across all models. In our cross-sectional analysis, barriers also emerged as a statistically significant covariate of MVPA [12]. In general, girls in this sample had higher perceived barriers, including obstacles related to physical comfort (i.e., sweating) and enjoyment (e.g., embarrassment and being chosen last for teams). Understanding the specific nature of these barriers - including whether they are "real" impedances versus reactionary explanations for why one is not active - warrants further investigation. Our data do suggest that, in this sample of girls, perceived barriers are distinct from the perception of self-efficacy, suggesting that the barriers that girls are responding to are not related to their PA abilities. A better understanding of how girls make decisions about the types and frequency of various activities might help to explain potential tools for overcoming such barriers.

This research expands upon our previous examination of the correlational associations found between levels of PA and factors drawn from our conceptual model based on a social ecological framework [12,13]. Our previous cross-sectional analysis, using baseline data from adolescents participating in the IDEA study showed that, for boys, self-efficacy, peer support, having PA equipment in the home and the average monthly temperature were all significantly related to MVPA, after adjusting for demographic characteristics. For girls, perceived barriers, distance to school and the walkability index were significantly related to MVPA, after adjustment for demographics. All relationships were in the expected direction.

Consistent with previous research, neither parent nor peer support as early exposures were shown to be predictive of PA among boys or girls [9]. Parental encouragement and family support has been associated with increased PA among males and females over time in some studies, [50,51] but not in others [48]. The role that parental and peer encouragement plays appears to vary depending on whether children enter puberty early or late, particularly for boys [48]. Similarly, we saw no evidence that the elements we assessed in the home and neighborhood environments were related to PA in our longitudinal analysis. The lack of association between environmental factors and activity levels may reflect the generally high socioeconomic status and potential for resources in our sample; the environment is adequate enough to not pose a barrier to activity. In a sample that is more restricted or limited in their resources, environmental factors may be more important in explaining behaviors [52]. Replicating this research in more diverse samples is warranted. In addition, future research examining how changes in the physical environment (whether through natural experiments or trials) might influence PA is warranted.

This manuscript expands the work presented by Patnode, which presented cross sectional findings using similar models [12]. Our longitudinal analysis was able to show differences in predictive factors by gender, which is important for intervention research. In addition, our longitudinal models were able to explain nearly a third of PA variance in boys and girls. The cross-sectional analysis conducted by Patnode et al. on part of this sample accounted for 25% and 15% among boys and girls, [12] respectively, compared to 30% and 24% in our models. Our inclusion of baseline levels of MVPA is likely responsible for the increased variance explained. Previous research, as noted in the introduction, has explained only 5% of the variance of PA among children [53]. Therefore, despite the limited significant independent predictors in our final models, a multilevel and longitudinal analysis was a better tool to understand the complexities of adolescent physical activity participation. However, we also acknowledge and recommend work on further development and refining of existing measures, particularly for youth transitioning from elementary to high school and beyond.

A number of limitations should be noted. The study sample was recruited from one metropolitan area within the Midwest and is predominantly white and of higher socioeconomic status, which may limit generalizability of the findings beyond this sample. Additionally, while a number of variables were included at multiple levels of influence, the specific items and scales captured in this study only represent a small number of potential influences on youth PA. The neighborhood assessment variables we used were modified from an adult survey. Future youth-oriented research should use a recently published youth-centered version of neighborhood environment questions [54]. Lastly, this study only examined the predictors of PA over 24 months. This time period may not have been long enough to capture any important changes or transitions that are happening within this population (developmental changes, moving from middle to high school, increasing independence and making decision about how to spend their time) that could ultimately influence participation in PA over time. More sophisticated analyses, including growth curve modeling with multiple time points, might better elucidate factors that relate to any increase or decrease in the trajectory of PA levels among children and adolescents.

Despite these limitations, this study fulfills many of the recommendations for future research offered by Craggs and colleagues [9] in their review of determinants in change in PA among children and adolescents. First, our study included an objective measurement of PA across a wide age range of children and adolescents. Second, our study included a comprehensive assessment of determinants within multiple levels, including both subjective perceptions as well as objective measures of the environment. Lastly, our study included a relatively large sample with an even distribution of boys and girls during an important developmental period.

## Conclusion

The lack of effectiveness of many interventions designed to increase PA among youth may, in part, be due to a poor understanding of the mechanisms responsible for behavior change [55]. Further prospective and intervention research, perhaps over longer periods of time, seems warranted if we are to design interventions that specifically target these potential mediators. In addition, it seems that more qualitative research to better understand the factors that influence children and adolescents to adopt and maintain higher levels of PA over time may prove beneficial.

## Competing interests

The authors declare that they have no competing interests.

## Support

Transdisciplinary Research in Energetics and Cancer (TREC) Initiative. Grant # 1U54CA116849-01 and from the National Heart, Lung and Blood Institute, Grant # R01HL085978. Supported by: Etiology of Childhood Obesity (ECHO) with funding from National Heart, Lung and Blood Institute, Grant # R01HL085978.

## Authors' contributions

MH was the lead author and provided analytic oversight and responsible for the content of the manuscript; CP provided additional content expertise and writing; JS provided content expertise and editing of document; KF conducted the analysis; LL was PI of the parent studies and contributed to the conception and editing of the document. All authors read and approved the final manuscript.
